# Glucose repression in *Saccharomyces cerevisiae*

**DOI:** 10.1093/femsyr/fov068

**Published:** 2015-07-23

**Authors:** Ömur Kayikci, Jens Nielsen

**Affiliations:** 1 Department of Biology and Biological Engineering, Kemivägen 10, Chalmers University of Technology, SE41296 Gothenburg, Sweden; 2 Novo Nordisk Foundation Center for Biosustainability, Chalmers University of Technology, SE41296 Gothenburg, Sweden; 3 Novo Nordisk Foundation Center for Biosustainability, Technical University of Denmark, DK2970 Hørsholm, Denmark

**Keywords:** carbon metabolism, Snf1 signaling, carbon catabolite repression

## Abstract

Glucose is the primary source of energy for the budding yeast *Saccharomyces cerevisiae*. Although yeast cells can utilize a wide range of carbon sources, presence of glucose suppresses molecular activities involved in the use of alternate carbon sources as well as it represses respiration and gluconeogenesis. This dominant effect of glucose on yeast carbon metabolism is coordinated by several signaling and metabolic interactions that mainly regulate transcriptional activity but are also effective at post-transcriptional and post-translational levels. This review describes effects of glucose repression on yeast carbon metabolism with a focus on roles of the Snf3/Rgt2 glucose-sensing pathway and Snf1 signal transduction in establishment and relief of glucose repression.

## Abstract (precis)

The role of Snf1 signaling in glucose repression and carbon metabolism in *Saccharomyces cerevisae*.

## Graphical abstract

**Graphical Abstract Figure.**
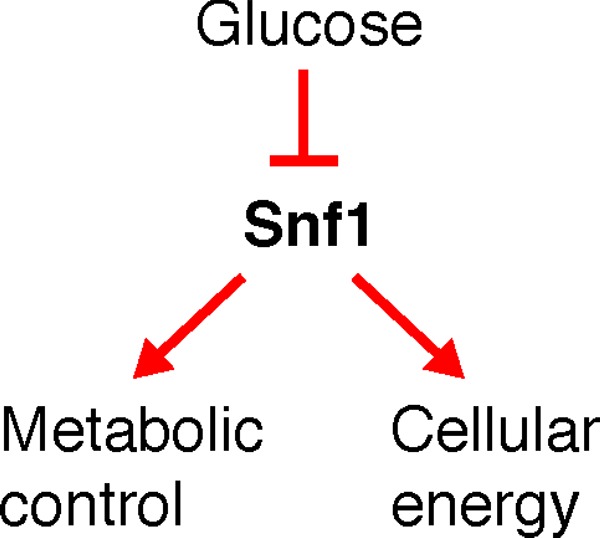
The role of Snf1 signaling in glucose repression and carbon metabolism in *Saccharomyces cerevisae*.

## INTRODUCTION

When glucose is accessible the yeast *Saccharomyces cerevisiae* prefers a fermentative metabolism despite presence of oxygen, and represses respiration, use of alternative carbon sources as well as gluconeogenesis (Klein, Olsson and Nielsen 1998; Rolland, Winderickx and Thevelein 2002). This repressive effect of glucose is transmitted to the cellular machinery by interlinked regulatory interactions and signaling pathways. These coordinative molecular activities mainly exert their effects at the transcriptional level, but they are also operative at post-transcriptional and post-translational levels.

Since *S. cerevisiae* primarily prefers glucose as a carbon source, sensing of extracellular and metabolized glucose levels is important for coordination of yeast carbon metabolism. Yeast cells adjust diverse cellular activities according to glucose levels detected extra- and intracellularly. The Snf3/ Rgt2 signaling pathway is a sensory cascades present in yeast for detecting extracellular glucose levels (Kaniak *et al.* 2004). Through this pathway, the cell can sense extracellular glucose levels and use this to regulate glucose uptake, and hereby trigger glucose repression. The Snf1 protein kinase signaling is central to functionality of glucose repression and to balance cellular energy levels. Snf1 kinase has a dual role in glucose repression, both as an activator and as a repressor. High glucose concentrations render Snf1 inactive which leaves the transcription factor Mig1 non-phosphorylated and hence being present in the nucleus where it exerts repression, together with Ssn6/ Tup1 complex, of genes involved in the utilization of alternative carbon sources (Gancedo 1998; Hedbacker and Carlson 2008). On the other hand, when glucose becomes limited Snf1 is active and phosphorylates Mig1 allowing release of glucose repression and expression of glucose-repressed genes. Beside its central role in regulating expression of glucose-repressed genes, Snf1 kinase has direct interaction with the transcriptional apparatus, and it has been implicated in chromatin modification (Kuchin, Treich and Carlson 2000). Moreover, as a part of its role in energy homeostasis Snf1 regulates metabolic enzymes involved in fatty acid metabolism and carbohydrate storage as well as a role affecting GCN4 translation and hence amino acid biosynthesis (Usaite *et al.* 2009; Hedbacker and Carlson 2010; Zhang *et al.* 2011).

Snf1 is the yeast AMP-activated kinase (AMPK), a regulatory kinase that is highly conserved in eukaryal cells. Understanding its function and regulation is therefore of broad and general interest, and much knowledge about AMPK has been acquired through the use of *S. cerevisiae* as a model organism (Petranovic and Nielsen 2008; Petranovic *et al.* 2010). Understanding glucose repression is, however, also of utmost importance for the development of biotechnological processes where it is desirable to redirect the carbon fluxes towards products of interests (Chen and Nielsen 2013; Dai *et al.* 2015; Li and Borodina 2015). Due to the high relevance of glucose repression in yeast, we therefore here review key pathways involved in this complex process in yeast.

### Snf3/Rgt2 signaling

**Figure 1.**
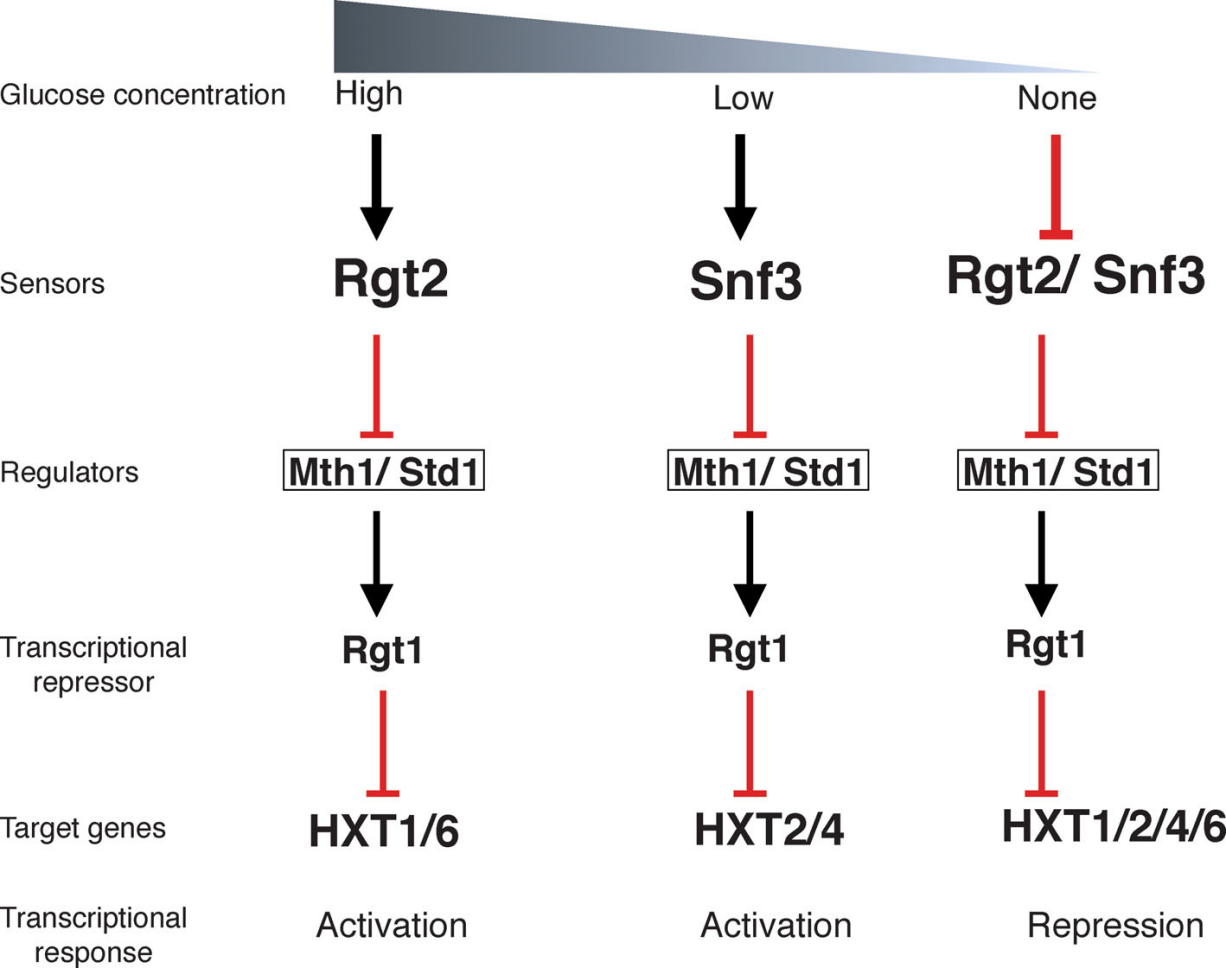
Snf3/ Rgt2 glucose-sensing pathway. Snf3/ Rgt2 glucose-sensing pathway dictates expression levels of hexose transporter (HXT) genes for optimal uptake of glucose at various concentrations.

Snf3/Rgt2 signaling pathway mainly senses varying concentrations of available glucose in the environment. The Snf3 and Rgt2 sensors belong to the HXT (HeXose transporters) gene family together with the Hxt1–17 and Gal2 proteins that all but Hxt12 can transport glucose (and other hexoses) but each with a different affinity for glucose (Wieczorke *et al.* 1999). Although structurally similar to hexose transporters, Snf3 and Rgt2 cannot transport glucose (Özcan, Dover and Johnston 1998). Snf3 senses low levels of extracellular glucose, while Rgt2 detects high levels of the sugar (Ozcan and Johnston 1999; Ozcan 2002). Intracellular signals generated upon detecting accessible amounts of glucose coordinate transcriptional regulation and expression of Hxt proteins (Fig. 1). Low-affinity transporters, such as Hxt1, are expressed and activated when glucose is abundant while under such conditions expression of high-affinity transporters, such as Hxt7, are repressed (Ozcan 2002). Snf3 and Rgt2 are also likely to sense internal-to-external ratio of glucose concentrations to adjust glucose uptake and maintain intracellular glucose homeostasis (Karhumaa, Wu and Kielland-Brandt 2010). Regulation of transcriptional activity through the Snf3/Rgt2 pathway allows *S. cerevisiae* to finely coordinate glucose uptake in response to environmental availability of this hexose sugar. When extracellular glucose is sensed by Snf3 and Rgt2 transmembrane proteins, the membrane-attached type I casein kinases Yck1 and Yck2 are activated. These active kinases are required for degradation of Mth1 and Std1, two paralogous regulatory proteins recruited to the plasma membrane (Schmidt *et al.* 1999; Moriya and Johnston 2004). Although Mth1 and Std1 were thought to be directly phosphorylated by Yck1/2 kinases for degradation, more recent data show that Mth1 is degraded in the nucleus independent of Yck1/2 localization (Pasula *et al.* 2010). So, according to this finding the glucose sensors transmit the glucose signal to a yet unidentified protein to promote phosphorylation and degradation of Mth1. Upon phosphorylation, Mth1 and Std1 are targeted for ubiquitination and proteosome degradation (Spielewoy *et al.* 2004). Furthermore, when glucose is abundant, Mig1 represses MTH1 expression to maintain glucose repression of the *HXT* genes. In contrast, as Std1 is being degraded its expression is increased to ensure efficient expression of the *HXT* genes when glucose is exhausted (Kim and Johnston 2006). Upon degradation of Mth1 and Std1, protein kinase A hyperphophorylates and dislodges Rgt1, transcriptional repressor of glucose-induced genes, from DNA (Palomino, Herrero and Moreno 2006). Glucose-induced translocation of PKA to the nucleus allows hyperphosphorylation of Rgt1 (Griffioen *et al.* 2000; Kim and Johnston 2006; Roy *et al.* 2013, 2014). This allows for expression of HXT genes and optimal uptake of glucose. Depletion of glucose renders Mth1 and Std1 available for Rgt1 interaction, which conceals PKA phosphorylation sites on Rgt1 and as a result the repressor remains bound to promoters suppressing expression of HXT genes when glucose is unavailable (Flick *et al.* 2003). Snf1 phosphorylation of Rgt1 triggers the repressor activity of Rgt1 and its propensity to bind DNA (Palomino, Herrero and Moreno 2006). This interaction between Rgt1 and Snf1 kinase is critical for graded derepression of HXT expression and plays an important role in overall glucose repression.

### SNF1 signaling

**Figure 2.**
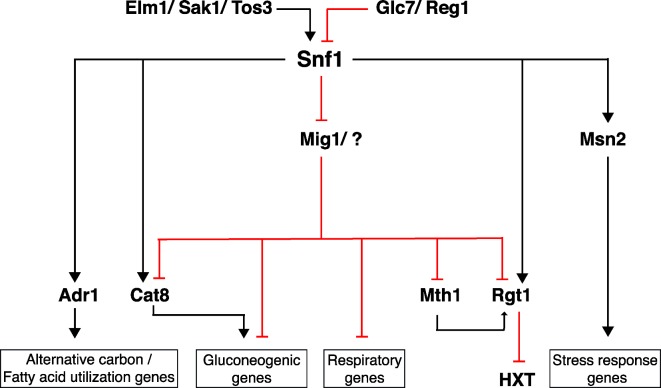
The Snf1 protein kinase is central player in glucose repression pathway. The Snf1 protein kinase regulates glucose repression at transcriptional level by activating or inactivating expression of gluconeogenic genes and genes involved in respiration.

When glucose in the environment is exhausted, yeast cells switch their metabolism from fermentation to respiration, and activate mechanisms and components for utilization of alternative carbon sources. This usually occurs in the late exponential phase of a batch culture, where the cells are often referred to as being ‘derepressed’. Snf1 plays a central role in this metabolic shift by regulating a range of repressors and activators. Availability of glucose regulates activity of this central player in glucose repression. During growth on optimal glucose levels, Snf1 is inactive and excluded from the nucleus. This allows for a major downstream target of Snf1 the transcription repressor Mig1 to be non-phosphorylated and in the nucleus. Conversely, a drop in the glucose concentration (to about 0.2%) activates Snf1 which in turn phosphorylates and deactivates Mig1 relieving glucose repression (Fig. 2) (Piskur and Compagno 2014).

The yeast Snf1 is a widely conserved serine/theronine kinase in eukaryotes required for cellular energy homeostasis. Like the mammalian AMPK, Snf1 kinase complex in *S. cerevisiae* has a heterotrimeric structure with an alpha catalytic subunit (Snf1), a gamma regulatory subunit (Snf4) and one of three beta subunits (Sip1, Sip2, Gal83) (Carlson, Osmond and Botstein 1981; Amodeo, Rudolph and Tong 2007). When glucose levels are high, Snf1 kinase complex is inactive due to autoinhibition as a result of interaction between the N-terminal catalytic domain and C-terminal regulatory domains of Snf1 (Celenza and Carlson 1989; Jiang and Carlson 1996; Ludin, Jiang and Carlson 1998; Leech *et al.* 2003). Low glucose concentrations remove this autoinhibition and promote interactions between Snf4 regulatory subunit and Snf1 catalytic subunits. Additionally, phosphorylation of the conserved residue at Thr^210^ is also required to activate Snf1 (McCartney and Schmidt 2001). In contrary to AMPK, Snf1 is not regulated by AMP but by ADP, which ensures a direct link between energy metabolism and this key regulator (Mayer *et al.* 2011).

Sak1, Elm1 and Tos3 are three protein kinases known to phosphorylate Thr^210^ in the Snf1 activation loop. These upstream kinases have overlapping functions since only deletion of all three abolishes Snf1 activation (Hong *et al.* 2003; Nath, McCartney and Schmidt 2003; Sutherland *et al.* 2003). So far, there has not been a report on how the glucose signal regulates the activating kinases of Snf1. Although each activating kinase contributes differently under different carbon source availability, Sak1 has the most stable interaction with Snf1; it is suggested to be the major activating kinase at conditions of growth on alternative carbon sources. The beta subunits also provide specificity for a particular upstream activating kinase under different conditions (McCartney, Rubenstein and Schmidt 2005). The three scaffolding (beta) subunits also regulate localization of Snf1 kinase. Sip1 directs Snf1 to vacuoles, while Sip2 keeps the enzyme in the cytoplasm and Gal83 play a role in nuclear localization of Snf1 (Vincent *et al.* 2001; Hedbacker, Hong and Carlson 2004). Each subunit has been indicated to have a distinct yet overlapping role in regulation of transcription and cellular metabolism. For example, Gal83 and Sip2 both play a major role in activating gluconeogenic genes and genes involved with the glyoxylate cycle while Sip1 is mainly involved in the regulation of genes associated with nitrogen metabolism (Zhang, Olsson and Nielsen 2010).

Another level of control is dephoshorylation of Snf1, which is carried out by Glc7/ Reg1 phosohotases (PP1) (Feng *et al.* 1991; Tu and Carlson 1995). This level of control has been hypothesized to be the main regulator of Snf1 activity since phosphorylation of Snf1 by upstream kinases (Sak1, Elm1 and Tos3) has not been affected by changes in glucose concentrations (Rubenstein *et al.* 2008). On the other hand, dephosphorylation of the kinase has been shown to be correlated with glucose availability in the environment (Sanz *et al.* 2000). Although activity of the Reg1/Glc7 phosphatase remained unchanged in response to glucose fluctuation in the environment, it has been hypothesized that the accessibility of Snf1 for dephosphorylation by the phosphatases changes (Rubenstein *et al.* 2008). Subsequent work, however, revealed that addition of glucose causes a rapid increase in Reg1 activity leading to inactivation of Snf1 (Castermans *et al.* 2012). This process could be part of the mechanism that transmits the information about the glucose level to Snf1 kinase. In addition to detecting the shift in glucose availability to establish glucose repression, Snf1 has also been suggested to monitor absolute glucose levels (Bendrioua *et al.* 2014). With the activation of Snf1 by ADP, there is, however, also a direct coupling of Snf1 activity with the energy status of the cell, and this is clearly related to the glucose metabolism.

### Transcriptional effects on carbon metabolism

Upon activation, Snf1 kinase interacts with a number of transcription factors, activating some while suppressing others. Mig1 transcriptional repressor is a major downstream target of Snf1 phosphorylation. When phosphorylated by Snf1, this transcription repressor is deactivated and released from DNA allowing expression of glucose-repressed genes, mainly genes for utilization of alternative carbon sources. Mig1 mediates suppression of glucose-repressed genes together with the general repressor complex Ssn6/Tup1 (Treitel and Carlson 1995; Lutfiyya *et al.* 1998). Mig1 also interacts with Hxk2 to suppress glucose-induced genes. Besides its metabolic role as glucose, kinase Hxk2 also affects transcriptional regulation of glucose-repressible genes. When glucose is abundant Hxk2 interacts with Mig1 at Ser^311^, a site that is also targeted by Snf1 for phosphorylation (Ahuatzi *et al.* 2007; Peláez, Herrero and Moreno 2010). By occupying this site, Hxk2 prevents Snf1 phosphorylation and hence removal of Mig1 from the nucleus. Furthermore, subsequent data suggest that Hxk2 is phosphorylated and dephosphorylated at Ser^14^ by Snf1 and Reg1/Glc7, respectively. Phosphorylation of Hxk2 prevents its nuclear localization and hence its interaction with transcription factors (Fernández-García *et al.* 2012).

Cat8 is another transcription factor regulated by Snf1 activation and required for gluconeogenesis and survival on alternative carbon sources (Fig. 2). Snf1 controls activity of this transcription factor at two levels. While removal of Mig1 repression by Snf1 allows for upregulation of CAT8 expression, Snf1 phosphorylation of Cat8 triggers its activation (Hedges, Proft and Entian 1995; Randez-Gil *et al.* 1997). Cat8 derepresses target genes by binding to carbon source-responsive elements (CSREs) in upstream regions of these genes (Young *et al.* 2003; Roth, Kumme and Schüller 2004). Key gluconeogenic genes as well as genes involved in the glyoxylate cycle and utilization of non-fermentable carbon sources, including *FBP1*, *MLS1* and *ICL1*, depend on Cat8 for their transcriptional regulation (Randez-Gil *et al.* 1997; Tachibana *et al.* 2007; Biddick, Law and Young 2008; Weinhandl *et al.* 2014). Recently, *Znf1* was identified as a transcription factor also involved in regulation of gluconeogenesis and the glyoxylate cycle, but it is not known if Snf1 regulates this transcription factor (Tangsombatvichit *et al.* 2015).

Another major yet indirect target of Snf1 is the Adr1 transcriptional activator. Under glucose-depleted conditions, Adr1 is important for ethanol utilization and fatty acid metabolism (Fig. 2). Adr1 activity is triggered by Snf1-mediated dephosphorylation and also Snf1 regulates chromatin binding of Adr1 when glucose is scarce (Young, Kacherovsky and Van Riper 2002; Schüller 2003; Ratnakumar *et al.* 2009; Turcotte *et al.* 2010). Under repressing conditions though Reg1/Glc7 phosphatases inhibit chromatin binding of Adr1 (Dombek, Kacherovsky and Young 2004). Moreover, Adr1 affects DNA binding of Cat8 (Tachibana *et al.* 2005; Biddick, Law and Young 2008). Like Cat8, Adr1 also binds to CSREs, and in fact they both target key genes for derepression. For example, Adr1 and Cat8 trigger *ADH2* transcription synergistically by binding at the upstream activation sites (Verdone *et al.* 2002; Tachibana *et al.* 2005). This binding is required for maximal expression of the *ADH2* gene product of which, alcohol dehydrogenase 2, is glucose repressed and required for ethanol catabolism (Walther and Schüller 2001). *ACS1*, acetyl CoA synthase, is another locus which complete derepression, requires Adr1 and Cat8 working synergistically (Kratzer and Schüller 1997). Adr1 coordinates metabolic activities mainly important for acetyl-CoA and NADH generation from alternative carbon sources, such as lipids (Young *et al.* 2003). Transcription of *FOX2*, a multifunctional enzyme involved in fatty acid degradation, also depends on Adr1 (Ratnakumar and Young 2010; Turcotte *et al.* 2010). Besides coordination of transcriptional factors, Snf1 regulates gene expression by chromatin remodeling. Upon glucose depletion, Snf1 kinase activation of Cat8 and Adr1 plays a role in chromatin remodeling for proper expression of glucose-repressed genes (Agricola *et al.* 2004; Tachibana *et al.* 2005). For example, Adr1 restructures the promoter of the *ADH2* gene to ensure proper transcriptional activity (Di Mauro 2000; Verdone *et al.* 2002). Moreover, Snf1 mediates chromatin restructuring also directly via Gcn5 acetyltransferase, as in the case of ADY2 glucose-repressed gene (Abate *et al.* 2012). Snf1 directly interacts with and phosphorylates the histone acetyltranferase Gcn5 and triggers its histone acetyl transferase activity (Liu, Xu and Kuo 2010). Gcn5-mediated acetylation is critical for transcriptional activation of many stress-responsive genes. For example, binding of Adr1 activation sites depends on the acetylation state of nucleosomes (Verdone *et al.* 2002). Another way Snf1 exerts its effects on transcriptional activity is by directly interacting with RNAII-pol holoenzyme. In response to glucose limitation, Snf1 kinase physically interacts and phosphorylates the holoenzyme and hereby triggers the transcription process (Kuchin, Treich and Carlson 2000; Young *et al.* 2012).

### Post-transcriptional and -translational effects on metabolism

**Figure 3.**
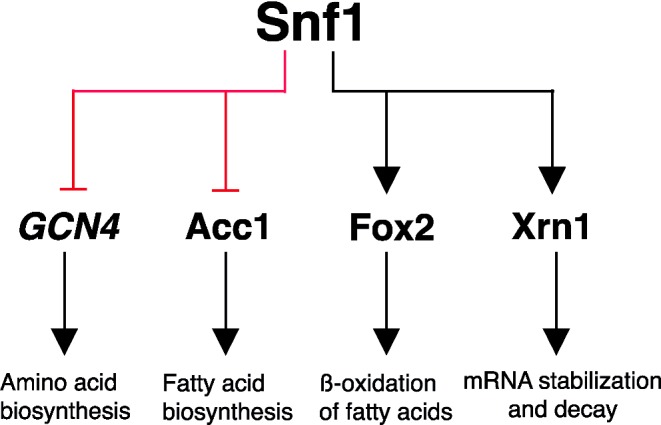
The Snf1 protein kinase regulates glucose repression at post-transcriptional and post-translational level. Prominent components of metabolic activity are direct targets of Snf1 kinase activity.

Dynamic interactions of Snf1 at post-transcriptional and post-translational levels are important for Snf1's role in balancing cellular energy levels when conditions are not favored. Snf1 achieves energy recalibration by inactivating energetically expensive cellular processes, such as amino acid and lipid biosynthesis, and meanwhile by upregulating programs, such as fatty acid oxidation, that generate energy. It is important to ensure coordinated regulation of carbon and nitrogen metabolism (Rødkær and Færgeman 2014), and under glucose starvation conditions Snf1 downregulates amino acid biosynthesis by inhibiting transcription and translation of *GCN4* (Fig. 3). Deletion of *SNF1* or inactivation of its kinase activity interestingly reveals significant induction in expression of genes mainly controlled by Gcn4 and required for amino acid generation (Shirra *et al.* 2008; Zaman *et al.* 2009). On the other hand, however, Snf1 has also been implicated in promoting translation initiation and activation of Gcn4 by two different mechanisms depending on the limiting conditions. When amino acid levels are limited Snf1 interacts with and activates Gcn2, which leads to increased phosphorylation of eIF2α for promoting Gcn4 translation initiation. Gcn2 and eukaryotic initiation factor 2α (eIF2α) are two regulators of Gcn4 translation initiation. Phosphorylation of eIF2α is critical for Gcn4 translation initiation. When Snf1 is active, it inhibits Sit4 and Glc7 phosphatases and hereby promotes the phosphorylation status of eIF2α resulting in Gcn4 translation initiation (Cherkasova, Qiu and Hinnebusch 2010). The differential effects Snf1 has on *GCN4* transcription and translation initiation under both carbon and nitrogen limitations emphasizes the interplay that Snf1 orchestrates between amino acid synthesis, carbon metabolism and cellular energy balance.

Snf1 coordinates carbon availability and cellular energy also through its effects on lipid metabolism. Fatty acid synthesis is energy demanding that has to be minimized during carbon source limitation (Klug and Daum 2014). Snf1 directly contributes to inhibition of this process by phosphorylating and inactivating acetyl carboxylase (Acc1) during energy deficiency (Fig. 3) (Woods *et al.* 1994; Shirra *et al.* 2001). This enzyme catalyzes the first rate-limiting step for *de novo* fatty acid biosynthesis, which is the formation of malonyl-Co A by carboxylation of acetyl CoA and removal of phosphorylation sites has been shown to result in an increased flux towards malonyl-CoA (Shi *et al.* 2014). In addition to inhibition of fatty acid synthesis, Snf1 promotes energy generation by stimulating degradation of fatty acids by peroxisome proliferation and β-oxidation. Fox2 is an additional factor associated with Snf1 in lipid degradation for energy synthesis (Usaite *et al.* 2009). This protein is a homodimeric multifunctional enzyme with 3-hydroxyacyl-CoA dehydrogenase and enoyl-CoA hydratese activities that allows Fox2 to have a wide substrate diversity for peroxisomal β-oxidation in yeast (Hiltunen *et al.* 2003). *In silico* studies have also identified Fox2 as a Snf1 phosphorylation target in lipid degradation (Ptacek *et al.* 2005; Usaite *et al.* 2009). Besides minimizing energy consumption and upregulating energy supply pathways, under prolonged glucose limitation Snf1 phoshorylates the general stress-responsive transcription factor Msn2 and hereby contributes to an adaptive response to poor glucose levels (Fig. 2) (De Wever *et al.* 2005).

Genetic and biochemical evidence suggest that Snf1 also plays a role in post-transcriptional control of gene regulation. mRNA decay is an important regulatory mechanism that together with nutrient-responsive transcriptional programs allows for a swift adaptation to unpredictable changes in environmental conditions. Inhibition of Snf1 activity leads to a rapid degradation of Snf1-dependent transcripts, whereas constitutive activation of Snf1 increases the stability of the same transcripts even in the presence of glucose (Young *et al.* 2012). Ccr4, Dhh1 and Xrn1 are three Snf1-targeted proteins involved in glucose-induced mRNA decay. Deletion of any of these proteins causes increased stability of Snf1-dependent transcripts in glucose-rich media (Young *et al.* 2012). Xrn1 has been suggested to be particularly important since an *xrn1* mutant is not viable under conditions in which Snf1 is highly active (Haimovich *et al.* 2013; Braun and Young 2014). Moreover, an *xrn1 reg1* double-deletion mutant shows synthetic lethality implying how vital Xrn1 is under poor nutrient conditions when Snf1 is active. Xrn1 is a conserved 5’ -> 3’ exoribonuclease that is critical for transcription-coupled mRNA decay, a process that balances transcription rate and mRNA degradation rate. Xrn1 modulates synthesis and stability of transcripts for genes required at glucose-poor conditions in a Snf1-dependent manner. Snf1 phosphorylates Xrn1 on sites located at the C-terminus and phosphorylated Xrn1 increases the stability of mRNA synthesized from glucose-repressed genes (Fig. 3). Furthermore, Snf1 phosphorylation of Xrn1 is necessary for glucose-induced decay of the same transcripts when glucose is replenished (Braun and Young 2014). The direct effects of Snf1 on mRNA metabolism in response to carbon source shift highlight its regulatory role in modulation of glucose repression.

## CONCLUSIONS

Glucose repression involves regulation of a multitude of genes and proteins involved in carbon source utilization and energy generation, and Snf3/Rgt2 sensors and the Snf1 kinase are important for this mechanism. Since yeast cells adjust molecular and cellular activities in response to levels of available glucose, integrating the glucose signal to carbon metabolism is highly regulated. Optimal uptake of glucose starts with the induction of the Snf3/Rgt2 pathway that upregulates expression of hexose transporters best suited for the concentration of glucose in the environment. Snf1 plays a pivotal role in orchestrating the effects of glucose on carbon metabolism. Inactive Snf1, in presence of glucose, mediates transcriptional repression of a multitude of genes including gluconeogenic genes, genes involved in respiration and utilization of non-fermentable carbon sources. Conversely, once activated upon glucose limitation Snf1 phosphorylates a number of proteins to mediate glucose derepression and activation of mechanisms required for energy generation from alternative carbon sources.

Although studies done so far indicate highly interactive and intricate roles Snf1 plays in regulating glucose repression and its effect on carbon metabolism, still much remains to be elucidated; for example, it is not known how Snf1 is inactivated in response to presence of excess glucose, and how Snf1 kinase interacts with other (known and unknown) regulators to control glucose repression.

***Conflict of interest.*** None declared.
